# Mapping artificial intelligence in problem-based and case-based medical education: a bibliometric analysis (2019–2026)

**DOI:** 10.3389/fmed.2026.1901004

**Published:** 2026-07-30

**Authors:** Qingyuan Tan, Yukui Ma, Jichun Zhao, Huanrui Hu

**Affiliations:** 1 West China Centre of Excellence for Pancreatitis, Institute of Integrated Traditional Chinese and Western Medicine, West China Hospital, Sichuan University, Chengdu, China; 2 Division of Vascular Surgery, Department of General Surgery, West China Hospital, Sichuan University, Chengdu, China

**Keywords:** artificial intelligence - AI, bibliometric analysis, case-based learning (CBL), ChatGPT, clinical reasoning, large language models (LLM), medical education - graduate, PBL (problem based learning) model

## Abstract

**Introduction:**

Problem-based learning (PBL) has been a cornerstone of medical education since its introduction at McMaster University in the 1960s. Since the public release of ChatGPT in November 2022, artificial intelligence (AI) tools have increasingly been applied to PBL and case-based learning (CBL) contexts, yet the research landscape at this intersection remains poorly characterized. This study aimed to map the growth trajectory, thematic structure, and collaboration networks of AI-PBL/CBL research from 2019 to 2026.

**Methods:**

A comprehensive search of Scopus and Web of Science was conducted on June 2, 2026, combining AI-related terms with PBL/CBL frameworks and medical education contexts. Using a PRISMA-guided bibliometric review workflow, 1,616 records were identified; after deduplication and eligibility screening, 735 unique publications (2019–2026, original articles, reviews, conference papers, and other eligible indexed document types) were included. Bibliometric analyses employed VOSviewer for network visualization (keyword co-occurrence, co-authorship, co-citation), CiteSpace for citation burst detection, and Bibliometrix for thematic mapping, three-field plot, and factorial analysis.

**Results:**

Publication output grew from 25 papers in 2022 to 254 in 2025, with 206 papers indexed by June 2, 2026. The United States (*n* = 70) and China (*n* = 64) led publication volume. Keyword co-occurrence analysis identified four thematic clusters: a central AI-focused cluster, a medical education and clinical reasoning cluster, a nursing and simulation-oriented cluster, and an educational technology cluster. Kung et al.'s 2023 study evaluating ChatGPT's performance on the USMLE was the most frequently co-cited reference (62 co-citations, betweenness centrality = 0.11). Thematic mapping positioned machine learning as a motor theme, while clinical reasoning, medical education, and self-directed learning appeared in the basic themes quadrant. The country/region collaboration network comprised 33 countries/regions and was led by the United States and China, although collaboration patterns remained uneven across regions.

**Conclusion:**

To our knowledge, this is the first bibliometric study specifically focused on the intersection of AI technologies with PBL/CBL in health professions education. The findings reveal rapid growth after 2023, four distinct but interconnected research clusters, and a collaboration network led by the United States and China, with uneven regional participation. These results may inform curriculum design and research priorities in AI-enhanced medical education.

## Introduction

Problem-based learning (PBL) has been a cornerstone of medical education since its introduction at McMaster University in the 1960s, emphasizing self-directed learning, clinical reasoning, and collaborative problem-solving (1). Over the past five decades, PBL has been widely adopted across medical curricula worldwide, with systematic reviews demonstrating its effectiveness in developing long-term knowledge retention and clinical reasoning skills (2, 3).

Since the public release of ChatGPT in November 2022, AI tools, particularly large language models (LLMs), have increasingly been discussed in relation to case generation, learner feedback, and clinical reasoning support in PBL-based medical education (4). LLMs can generate clinical cases, simulate patient interactions, provide feedback on diagnostic reasoning, and assist with literature synthesis, offering new capabilities for PBL workflows (5, 6). Studies have demonstrated that AI tools can pass medical licensing examinations (7, 8), generate differential diagnoses (9), and serve as virtual standardized patients (10), suggesting their potential to augment certain PBL components.

However, the integration of AI into PBL raises questions about educational effectiveness and the potential erosion of foundational clinical skills (11). While AI may enhance learning efficiency, concerns persist regarding its impact on critical thinking development, knowledge retention, and the authenticity of clinical reasoning (12, 13). Medical educators need evidence-based guidance on incorporating AI tools into PBL curricula (14).

Despite the surge in AI-PBL publications, no bibliometric analysis has specifically focused on the intersection of AI technologies with PBL and case-based learning (CBL) methodologies. Existing reviews have broadly examined AI in medical education (15, 16) or PBL effectiveness (2), but none have mapped the thematic structure and collaboration networks at this intersection. CBL was included alongside PBL in this analysis because these two pedagogical approaches share substantial conceptual overlap in medical education practice and are frequently discussed together in the literature; excluding CBL would risk omitting relevant studies that employ these frameworks interchangeably. Bibliometric analysis offers a quantitative approach to identifying research trends, collaborative networks, intellectual structures, and emerging frontiers (17).

This study aimed to: (1) characterize the growth trajectory and geographic distribution of AI-PBL/CBL research from 2019 to 2026; (2) identify major research themes and their evolution through keyword co-occurrence and thematic mapping; (3) map international and institutional collaboration networks; (4) identify the intellectual foundation through co-citation analysis; and (5) detect emerging research frontiers.

## Materials and methods

### Search strategy

For this descriptive bibliometric review/analysis, a comprehensive literature search was conducted on June 2, 2026, using Scopus (Elsevier) and Web of Science (WoS) Core Collection (Clarivate Analytics). Both databases were searched without language restrictions. The search strategy combined three concept clusters using the Boolean operator AND:

Cluster 1—AI-related terms: (‘artificial intelligence' OR ‘machine learning' OR ‘deep learning' OR ‘large language model' OR ‘LLM' OR ‘ChatGPT' OR ‘natural language processing' OR ‘generative AI' OR ‘GPT' OR ‘neural network').

Cluster 2—PBL/CBL frameworks: (‘problem-based learning' OR ‘PBL' OR ‘case-based learning' OR ‘CBL'). CBL was included alongside PBL because both are small-group, case-driven pedagogical approaches that share substantial conceptual and practical overlap in health professions education. In many curricula, PBL and CBL are used interchangeably or in hybrid formats, making it methodologically appropriate to capture both within a combined search strategy. Excluding CBL would risk omitting relevant studies that employ these frameworks interchangeably or refer to PBL variants under the CBL label. To reduce ambiguity from bare acronyms, the PBL/CBL term cluster was combined with a mandatory medical or health professions education context cluster; records were then screened against the eligibility criteria rather than excluded through a separate non-medical CBL category.

Cluster 3—Educational context: (‘medical education' OR ‘clinical education' OR ‘health professions education' OR ‘nursing education' OR ‘dental education' OR ‘medical student' OR ‘clinical reasoning').

Scopus raw search string (executed via Advanced Search): TITLE-ABS-KEY[(‘artificial intelligence' OR ‘machine learning' OR ‘deep learning' OR ‘large language model' OR ‘LLM' OR ‘ChatGPT' OR ‘natural language processing' OR ‘generative AI' OR ‘GPT' OR ‘neural network') AND (‘problem-based learning' OR ‘PBL' OR ‘case-based learning' OR ‘CBL') AND (‘medical education' OR ‘clinical education' OR ‘health professions education' OR ‘nursing education' OR ‘dental education' OR ‘medical student' OR ‘clinical reasoning')]. This search was conducted in the Title–Abstract–Keywords (TITLE-ABS-KEY) fields and retrieved 997 records, including 33 complete bibliographic fields (Citation information, Bibliographical information, Abstract & Keywords).

WoS raw search string (executed via Advanced Search): TS = (‘artificial intelligence' OR ‘machine learning' OR ‘deep learning' OR ‘large language model' OR ‘LLM' OR ‘ChatGPT' OR ‘natural language processing' OR ‘generative AI' OR ‘GPT' OR ‘neural network') AND TS = (‘problem-based learning' OR ‘PBL' OR ‘case-based learning' OR ‘CBL') AND TS = (‘medical education' OR ‘clinical education' OR ‘health professions education' OR ‘nursing education' OR ‘dental education' OR ‘medical student' OR ‘clinical reasoning'). This search was conducted in the Topic (TS) field, which encompasses Title, Abstract, Author Keywords, and Keywords Plus. A total of 619 records were retrieved and exported in Plain Text format with the ‘Full Record and Cited References' option.

**Figure 1:**
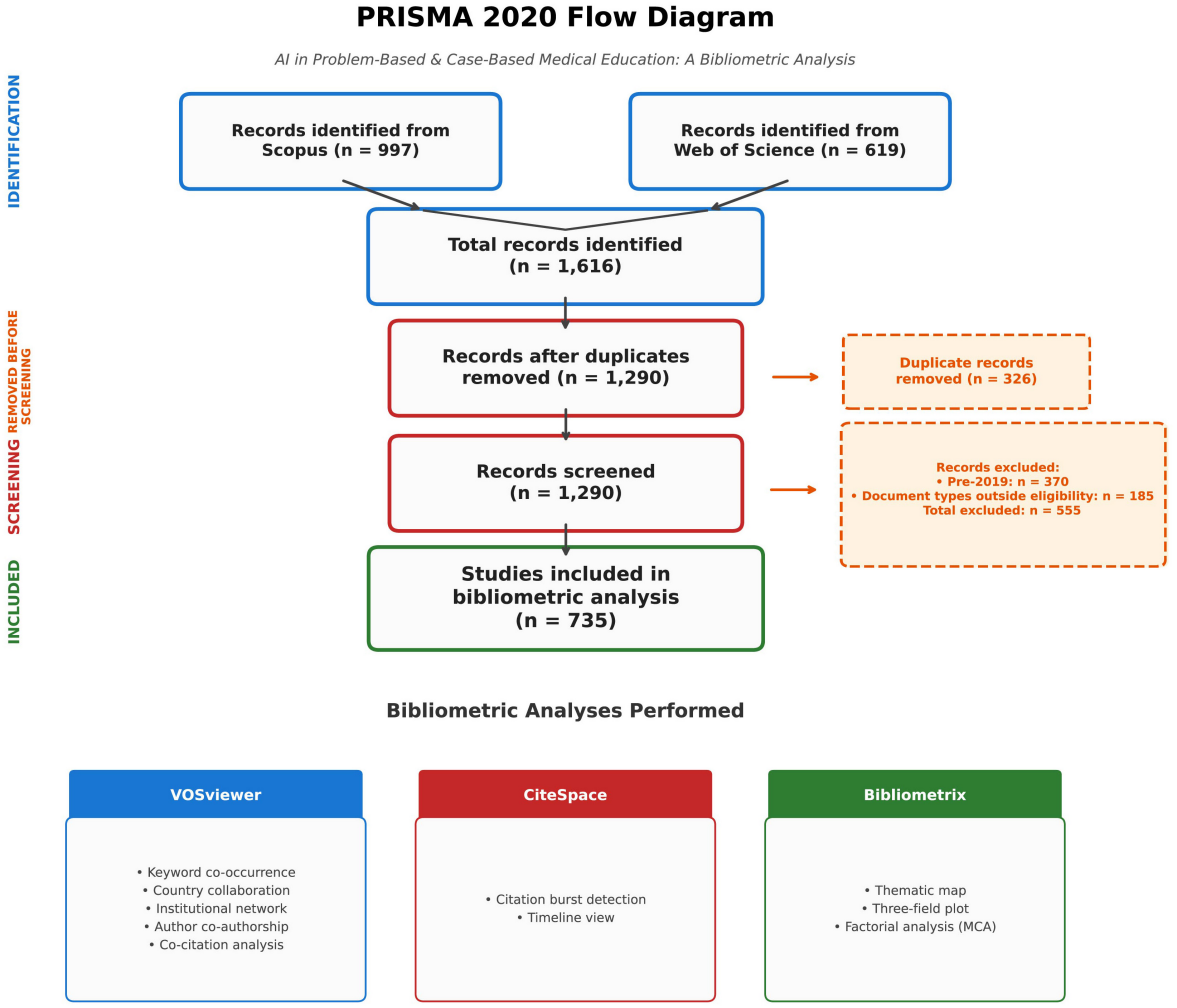
PRISMA 2020 flow diagram illustrating the literature identification, screening, and selection process for the bibliometric dataset.

Year (2019–2026) and document-type (article, review, conference paper) filters were applied during the screening stage (see Study Selection and Data Processing), consistent with the PRISMA 2020 flow diagram (Figure 1). The full search strategies are summarized in Supplementary Table S1. No language restrictions were applied at the search stage; however, because both Scopus and WoS are English-language-dominant databases, the retrieved literature is predominantly in English. Publications in other languages indexed with English titles/abstracts were included if they met all other criteria.

### Eligibility criteria

Publications were included if they: (a) were original articles, reviews (including systematic, scoping, and narrative reviews), or conference papers; (b) were published between January 1, 2019 and June 2, 2026; and (c) addressed the integration or application of AI technologies (including but not limited to machine learning, deep learning, natural language processing, large language models, and generative AI) within PBL or CBL contexts in health professions education (medicine, nursing, dentistry, pharmacy, and allied health). The 2019 starting date was selected to provide a balanced 4-year pre-ChatGPT baseline (2019–2022) and a 4-year post-ChatGPT period (2023–2026). Publications were excluded if they: (a) were editorials, letters, commentaries, errata, book reviews, or data papers; (b) addressed AI applications exclusively outside educational contexts (e.g., clinical diagnosis, drug discovery, or hospital operations without educational implications for PBL/CBL learners); or (c) discussed PBL/CBL without any substantive reference to AI technologies. Database-specific indexed records with inconsistent document-type labels after database merging were retained only if they met all eligibility criteria after manual screening.

### Study selection and data processing

Using PRISMA 2020 as a reporting framework for bibliographic identification and screening (18), the study selection process proceeded through four stages. (1) Identification: the initial search identified 997 records from Scopus and 619 from WoS (total: 1,616). (2) Duplicate removal: records were deduplicated by DOI matching when both Scopus and WoS records carried a DOI (approximately 82% of records). For records lacking a DOI (~18%), deduplication was performed by title-similarity comparison after normalizing for punctuation, case, and spacing (Levenshtein distance threshold of 0.85). This process removed 326 duplicate records, yielding 1,290 unique records. (3) Screening: the 1,290 records were screened against the eligibility criteria. Records published before 2019 (*n* = 370) and document types outside eligibility criteria (*n* = 185) were excluded, leaving 735 records (4). Inclusion: all 735 records that passed screening were included in the bibliometric analysis; no additional exclusions were made at the full-text review stage because bibliometric analysis captures publication metadata rather than study-level findings. Because this study is a descriptive bibliometric analysis of publication metadata rather than an intervention-effect systematic review, PICOS criteria were not used to define eligibility; instead, eligibility was operationalized through database source, year, document type, AI/PBL/CBL relevance, and health professions education context. The PRISMA 2020 checklist was completed as an adapted reporting aid and is available as Supplementary Material.

### Bibliometric analysis

Bibliometric analyses were conducted using three complementary tools. VOSviewer (version 1.6.20, Center for Science and Technology Studies, Leiden University, the Netherlands) was employed for network visualization with the following parameter configurations: (a) keyword co-occurrence network: unit of analysis = all keywords (author keywords plus index keywords), counting method = full counting, minimum occurrence threshold = 5, top 50 nodes by relevance displayed; (b) country co-authorship network: unit = countries, counting method = full counting, minimum document threshold = 3; (c) institutional co-authorship network: unit = organizations, minimum document threshold = 4; (d) author co-authorship network: unit = authors, minimum document threshold = 4; (e) co-citation analysis: unit = cited references, minimum citation threshold = 20. All networks were constructed using the association strength normalization method, and clusters were identified using the Leiden algorithm with default resolution parameter (resolution = 1.0). A thesaurus file (Supplementary Table S2) was created to standardize synonymous and variant terms before keyword co-occurrence analysis.

In the VOSviewer thesaurus file, spelling variants and capitalization variants were harmonized, but conceptually distinct constructs were retained separately; specifically, self-directed learning and self-regulated learning were not merged.

### Additional analyses

CiteSpace (version 6.3.R1, College of Computing and Informatics, Drexel University, USA) was used for citation burst detection and co-citation timeline visualization. CiteSpace parameters: time slicing = 2019–2026, years per slice = 1, node type = cited reference, selection criteria = g-index (k = 25), pruning = Pathfinder and pruning sliced networks, burst detection parameters: γ = 1.0, minimum duration = 1 year.

Bibliometrix (R package version 4.1.3, Department of Economics and Statistics, University of Naples Federico II, Italy) was run via the Biblioshiny web interface. Three analyses were performed: (a) Thematic map: field = author keywords (DE), clustering algorithm = Walktrap, number of words = 250, minimum cluster frequency = 5, number of labels per cluster = 3, quadrant thresholds defined by median centrality and median density (19); (b) Three-field plot (Sankey diagram): fields = authors (AU), number of items = 20 per field, for the full 2019–2026 period; (c) Factorial analysis (Multiple Correspondence Analysis, MCA): field = author keywords (DE), number of terms = 50, dimensionality reduction to two principal dimensions. Thematic map quadrants were defined based on Callon's centrality and density metrics (19): motor themes (high centrality, high density), basic themes (high centrality, low density), niche themes (low centrality, high density), and emerging or declining themes (low centrality, low density).

CiteSpace co-citation and burst analyses were conducted using WoS cited-reference records. Scopus records were included in the merged descriptive dataset but were not merged into the CiteSpace co-citation network because Scopus and WoS cited-reference exports differ in structure and field standardization.

### Software and reproducibility

**Figure 2:**
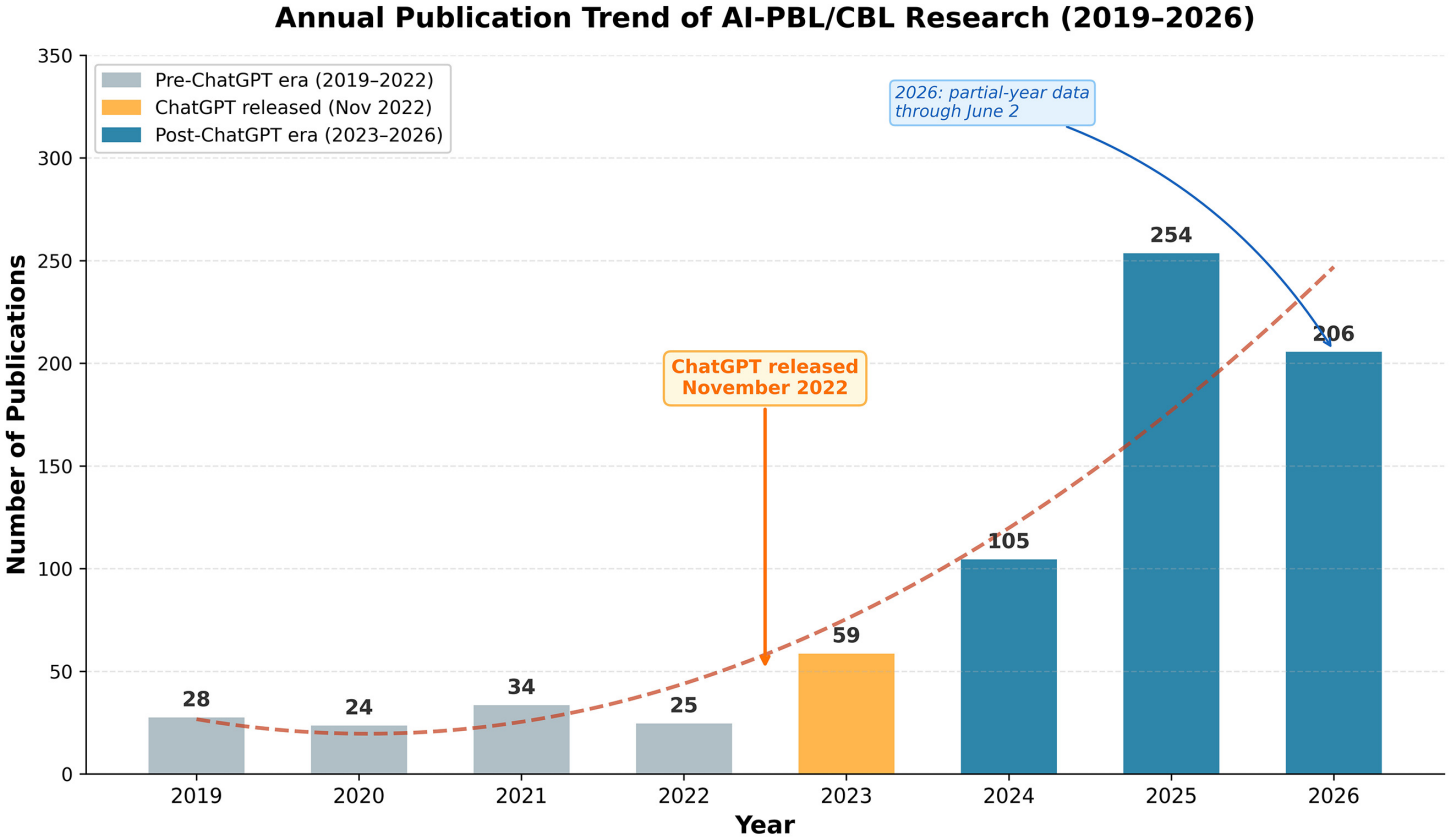
Annual publication trend of AI-PBL/CBL research (2019–2026). The dashed line represents a second-degree polynomial trend fit applied to complete years (2019–2025); the 2026 partial-year value was not included in trend fitting. The vertical annotation marks the public release of ChatGPT in November 2022. 2026 data are partial-year records retrieved through June 2, 2026.

All bibliographic data exported from Scopus and WoS were processed using custom Python scripts (version 3.13; pandas, NumPy) for data cleaning, deduplication, descriptive statistics, and figure generation (Figures 1, 2). The derived bibliometric dataset, processing scripts, VOSviewer thesaurus file, and analysis outputs are available from the corresponding author upon reasonable request and will be deposited in a public repository (Zenodo) upon manuscript acceptance. The use of two independent bibliographic databases (Scopus and WoS) with complementary coverage serves as a cross-validation mechanism: trends consistent across both databases are considered robust, while database-specific findings are noted as such in the Results.

## Results

### Publication output and growth trends

A total of 735 publications were included in the final analysis, comprising 527 articles (71.7%), 83 reviews (11.3%), 49 conference papers (6.7%), and 76 other document types (10.3%) (Table 1). Publication output showed rapid growth over the study period (Figure 2). During the pre-ChatGPT era (2019–2022), annual publications remained relatively stable, ranging from 24 to 34 papers (mean: 27.8 ± 4.2). Following the public release of ChatGPT in November 2022, output accelerated: 59 papers were published in 2023 (136% increase from 2022), 105 in 2024 (78% increase), and 254 in 2025 (142% increase)—a 4.3-fold increase from 2022 to 2025. By the search date of June 2, 2026, 206 papers had been indexed in 2026; because these records cover only January 1 to June 2, 2026, they are reported as partial-year data and were excluded from trend fitting, with no full-year extrapolation made.

**Table 1:** Basic characteristics of included literature.

Characteristic	Category	Value
Total publications	—	735
Year range	2019–2026	—
Scopus	Records identified	997
Web of science	Records identified	619
	Duplicate records removed	326
	Records screened	1,290
Article		527 (71.7%)
Review		83 (11.3%)
Conference paper		49 (6.7%)
Other^†^		76 (10.3%)
2019		28
2020		24
2021		34
2022		25
2023		59
2024		105
2025		254
2026 (through June 2)		206

^†^“Other” includes early access articles, online-first records, short surveys, and database-specific indexed records whose document-type classification varied between Scopus and WoS after merging. All 76 records in this category met the study's eligibility criteria.

### Journal distribution

The 735 publications were distributed across more than 200 academic journals. BMC Medical Education was the most prolific journal with 74 publications (10.1% of total), followed by JMIR Medical Education (*n* = 24, 3.3%), Medical Teacher (*n* = 19, 2.6%), Medical Science Educator (*n* = 18, 2.4%), and Nurse Education in Practice (*n* = 18, 2.4%) (Table 2A). These top five journals accounted for 20.8% of all publications. The Journal of Medical Internet Research had the highest impact factor among the top 10 journals (IF = 7.1).

**Table 2A:** Top 10 most productive journals in AI-PBL/CBL research.

Rank	Journal	Publications	IF (2024)	Country
1	BMC Medical Education	74	3.6	UK
2	JMIR Medical Education	24	3.2	Canada
3	Medical Teacher	19	4.1	UK
4	Medical Science Educator	18	1.0	USA
5	Nurse Education In Practice	18	3.4	UK
6	J Med Internet Research	13	7.1	Canada
7	Adv Med Educ Pract	12	1.8	UK
8	BMC Nursing	10	2.6	UK
9	Diagnosis	8	2.1	Germany
10	Nurse Education Today	8	3.6	UK

Top 10 journals.

### Country and institutional collaboration

**Figure 3:**
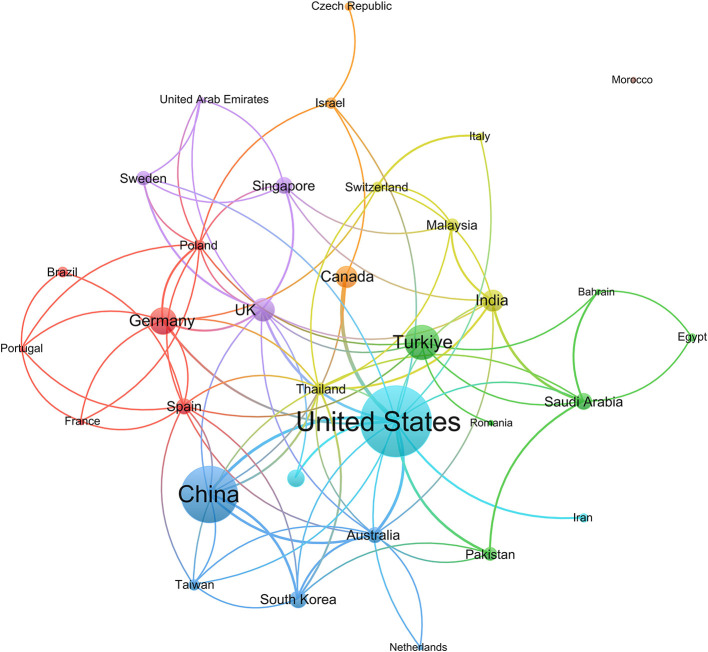
International country/region co-authorship network of AI-PBL/CBL research. Only countries/regions with at least three publications are displayed. Node size reflects publication count, link thickness reflects co-authorship frequency, and colors represent collaboration clusters identified by VOSviewer. Country/region names were harmonized for display where appropriate.

Country/region co-authorship network analysis identified several collaboration clusters comprising 33 countries/regions with three or more publications (Figure 3). The United States was the most productive country/region (*n* = 70 publications, 9.5%), followed by China (*n* = 64, 8.7%), Turkey (*n* = 34, 4.6%), Germany (*n* = 19, 2.6%), and Canada (*n* = 17, 2.3%) (Table 2B). The network showed three broad collaboration patterns: a European cluster involving Germany, the United Kingdom, France, Spain, and Poland; a North American–Asia-Pacific pattern led by the United States and China; and a Middle Eastern and transregional cluster centered on Turkey and linked with Saudi Arabia, Egypt, Pakistan, and India. Morocco was the only country/region among the 33 displayed nodes with no international co-authorship links (total link strength = 0), suggesting limited integration into the global collaboration network.

**Table B:** Top 10 countries/regions.

Rank	Country	Publications	% Of total
1	USA	70	9.5
2	China	64	8.7
3	Turkey	34	4.6
4	Germany	19	2.6
5	Canada	17	2.3
6	India	15	2.0
7	Japan	14	1.9
8	South Korea	12	1.6
9	UK	11	1.5
10	Sweden	10	1.4

### Institutional networks

**Figure 4:**
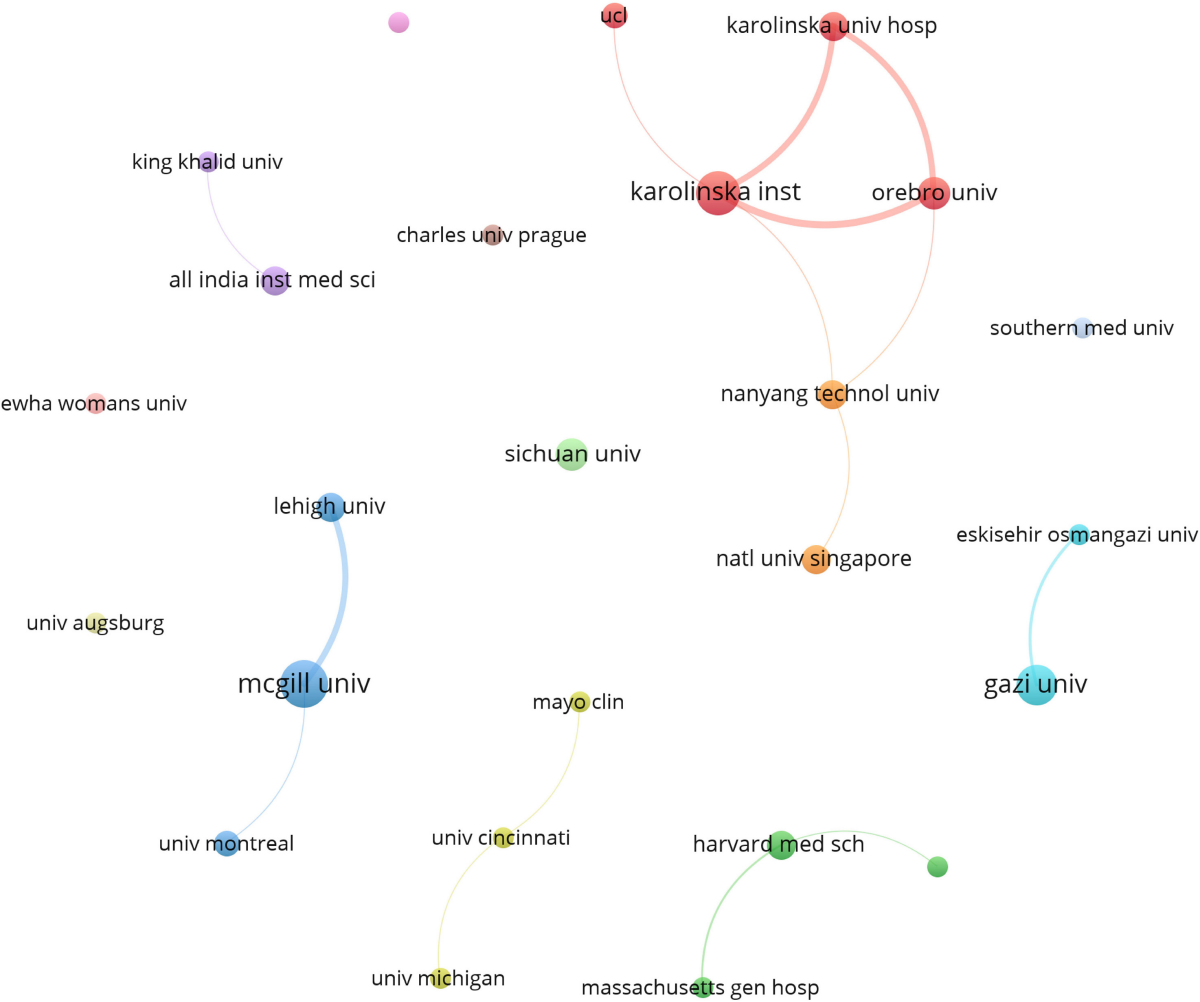
Institutional collaboration network (≥4 publications per institution).

Institutional co-authorship analysis (minimum threshold: 4 publications) identified 27 institutions forming six major collaborative clusters (Figure 4). Karolinska Institutet (Sweden) emerged as the most centrally connected institution, with a tightly integrated cluster including Karolinska University Hospital and Örebro University. Harvard Medical School formed a prominent North American cluster with Massachusetts General Hospital, Mayo Clinic, and the University of California San Francisco. McGill University formed a smaller collaborative cluster involving Université de Montréal and Lehigh University. The National University of Singapore and Nanyang Technological University formed a distinct Southeast Asian technology-oriented cluster.

### Author collaboration networks

**Figure 5:**
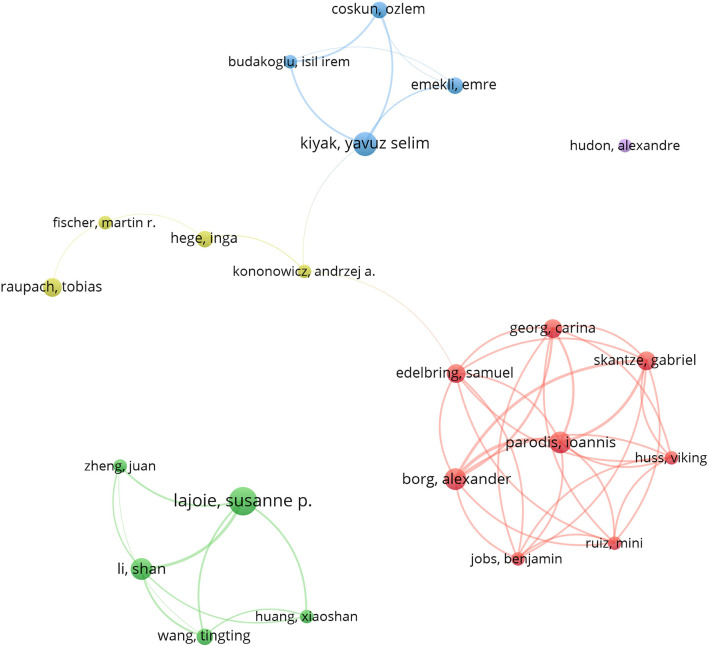
Author co-authorship network (≥4 publications per author).

Author co-authorship analysis (minimum threshold: 4 publications) identified 27 authors forming five distinct collaborative clusters (Figure 5). The largest cluster centered on Edelbring S. (Karolinska Institutet), comprising eight Swedish researchers with the most densely interconnected network. The second cluster, led by Lajoie S.P. (McGill University), included four Chinese researchers (Li S., Huang X., Zheng J., Wang T.), reflecting active North American–East Asian collaboration. The third cluster was anchored by Turkish researchers Kiyak Y.S. and Coşkun Ö. (Gazi University). Li S. and Lajoie S.P. were the most productive author-name strings with 12 publications each, followed by Hege I. (11 publications) (Table 2C). Several top-10 author strings with common Chinese surnames (Wang Y., Li J., Zhang Y., Li X.) likely aggregate multiple distinct researchers; these counts are therefore interpreted as author-name-string productivity rather than verified individual-level productivity, and this caveat is stated in the Table 2C footnote.

**Table C:** Top 10 authors.

Rank	Author	Publications	Primary institution	Country
1	Li S.	12	McGill University	Canada
2	Lajoie S.P.	12	McGill University	Canada
3	Hege I.	11	University of Augsburg	Germany
4	Wang Y.†	10	Multiple institutions	China
5	Raupach T.	9	University of Augsburg	Germany
6	Kononowicz A.A.	9	Jagiellonian University	Poland
7	Li J^†^	8	Multiple institutions	China
8	Zhang Y^†^	8	Multiple institutions	China
9	Zheng J.	7	McGill University	Canada
10	Li X^†^	7	Multiple institutions	China

^†^Author names are reported as database author-name strings. Common Chinese surnames may correspond to multiple distinct researchers; publication counts for these entries should therefore be interpreted as name-string-level estimates rather than verified individual-level productivity. The institution listed represents the most frequent affiliation in the dataset.

### Keyword co-occurrence and thematic clusters

**Figure 6:**
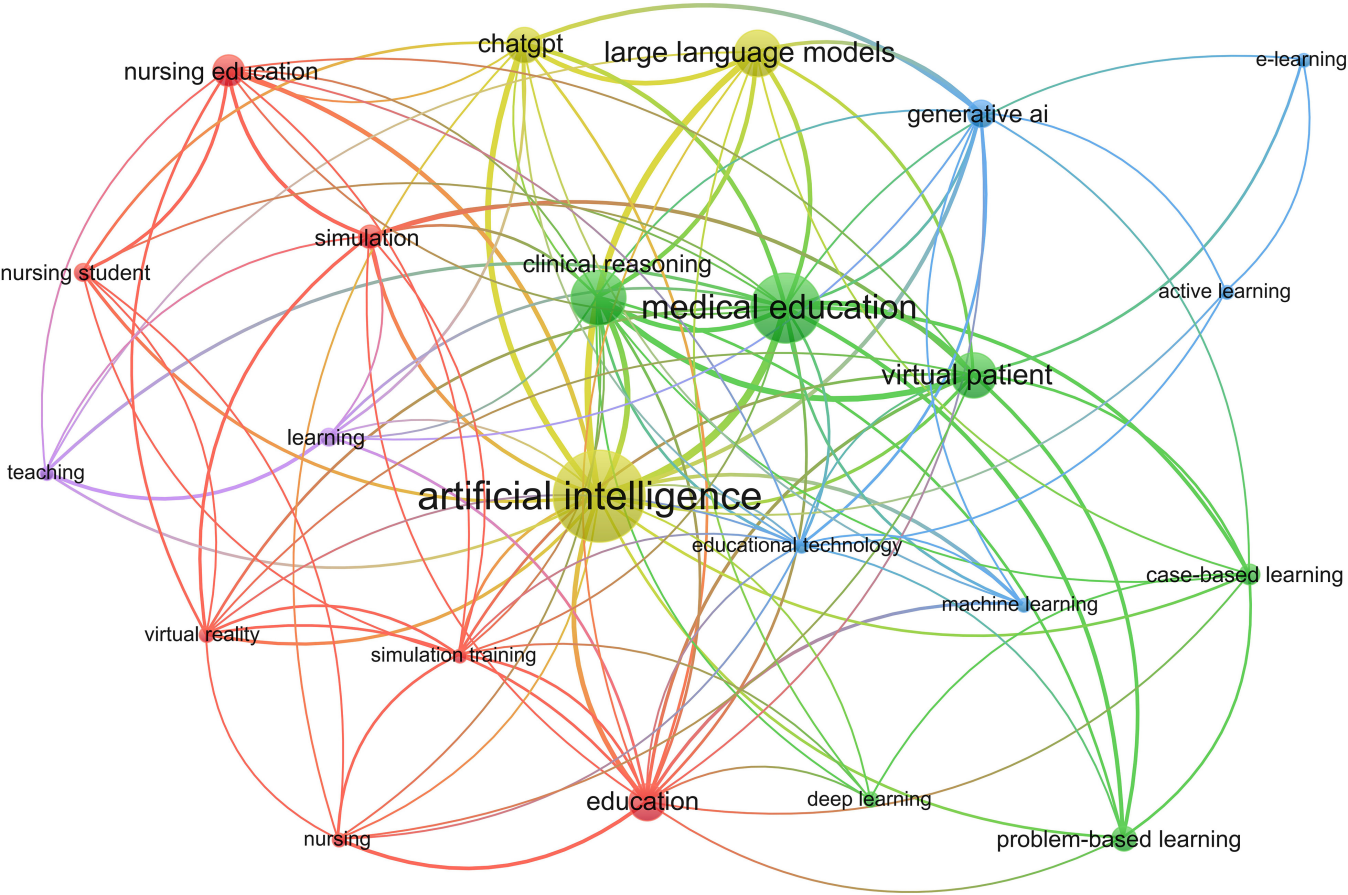
Keyword co-occurrence network (threshold ≥5, top 50 nodes). Colors represent thematic clusters identified by the Leiden algorithm.

Keyword co-occurrence analysis (minimum occurrence threshold: 5) revealed four major thematic clusters (Figure 6). The central AI-focused cluster was anchored by “artificial intelligence,” “ChatGPT,” and “large language models,” reflecting the recent expansion of generative AI and LLM-related research. A medical education and clinical reasoning cluster included “medical education,” “clinical reasoning,” “virtual patient,” “problem-based learning,” and “case-based learning,” indicating the pedagogical contexts in which AI tools are most frequently discussed. A nursing and simulation-oriented cluster was characterized by “nursing education,” “nursing student,” “simulation,” “simulation training,” and “virtual reality.” A fourth educational technology cluster included “generative AI,” “e-learning,” “active learning,” “educational technology,” and “machine learning.” Overall, “artificial intelligence” occupied a central bridging position, linking LLM-focused research with PBL/CBL, clinical reasoning, simulation, and health professions education.

### Thematic evolution and conceptual structure

**Figure 7:**
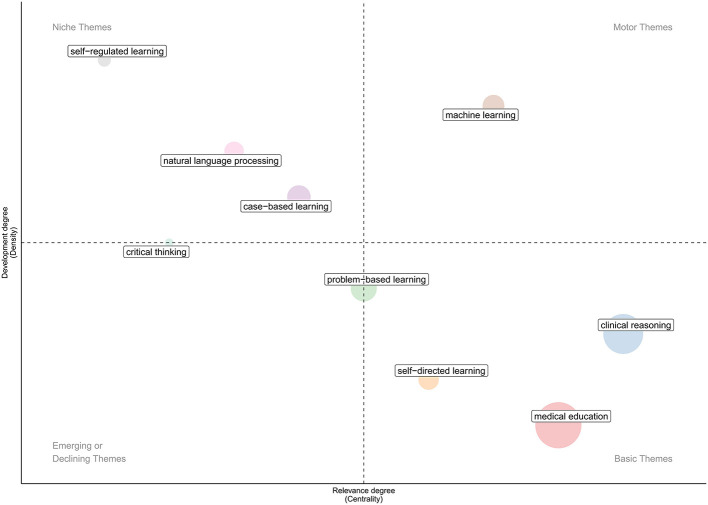
Thematic map based on author keyword analysis (Walktrap clustering). Quadrants: upper-right = motor themes; lower-right = basic themes; upper-left = niche themes; lower-left = emerging/declining themes.

**Figure 8:**
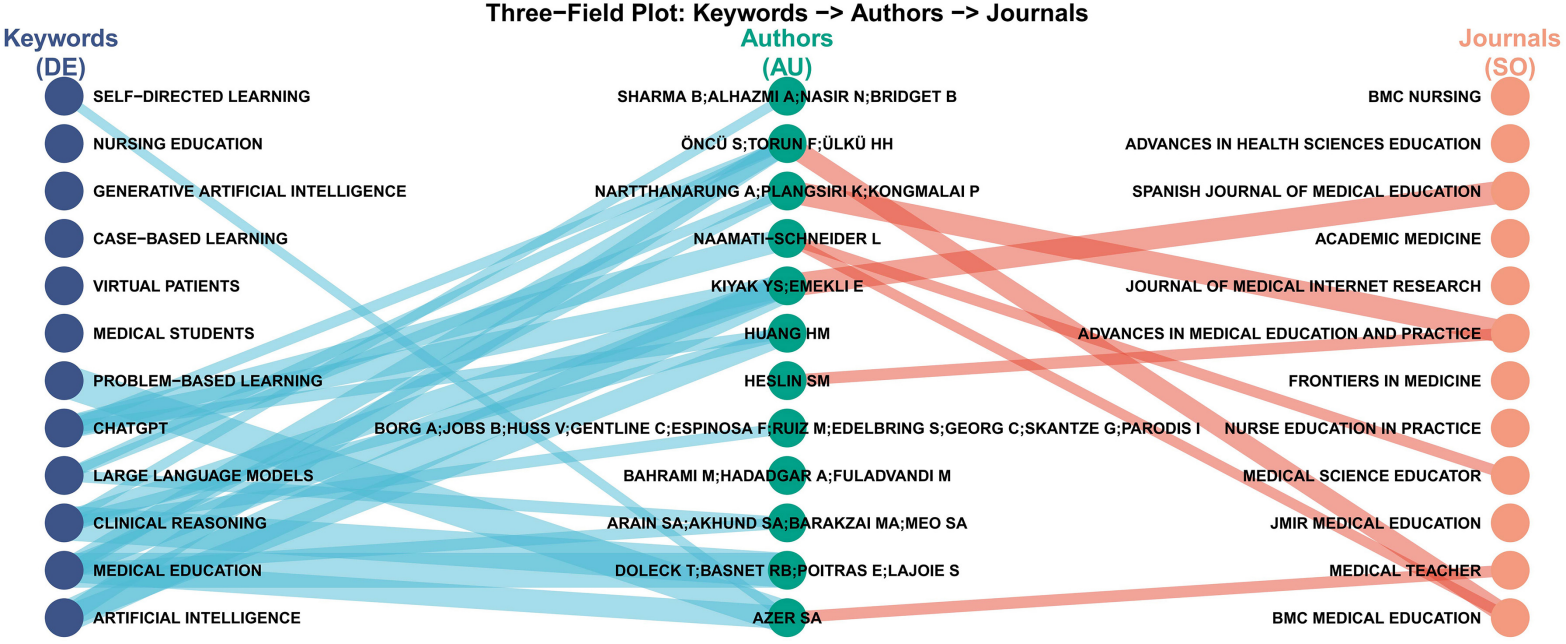
Three-field plot (Sankey diagram): keywords **(left)**97authors **(middle)**97journals **(right)**.

**Figure 9:**
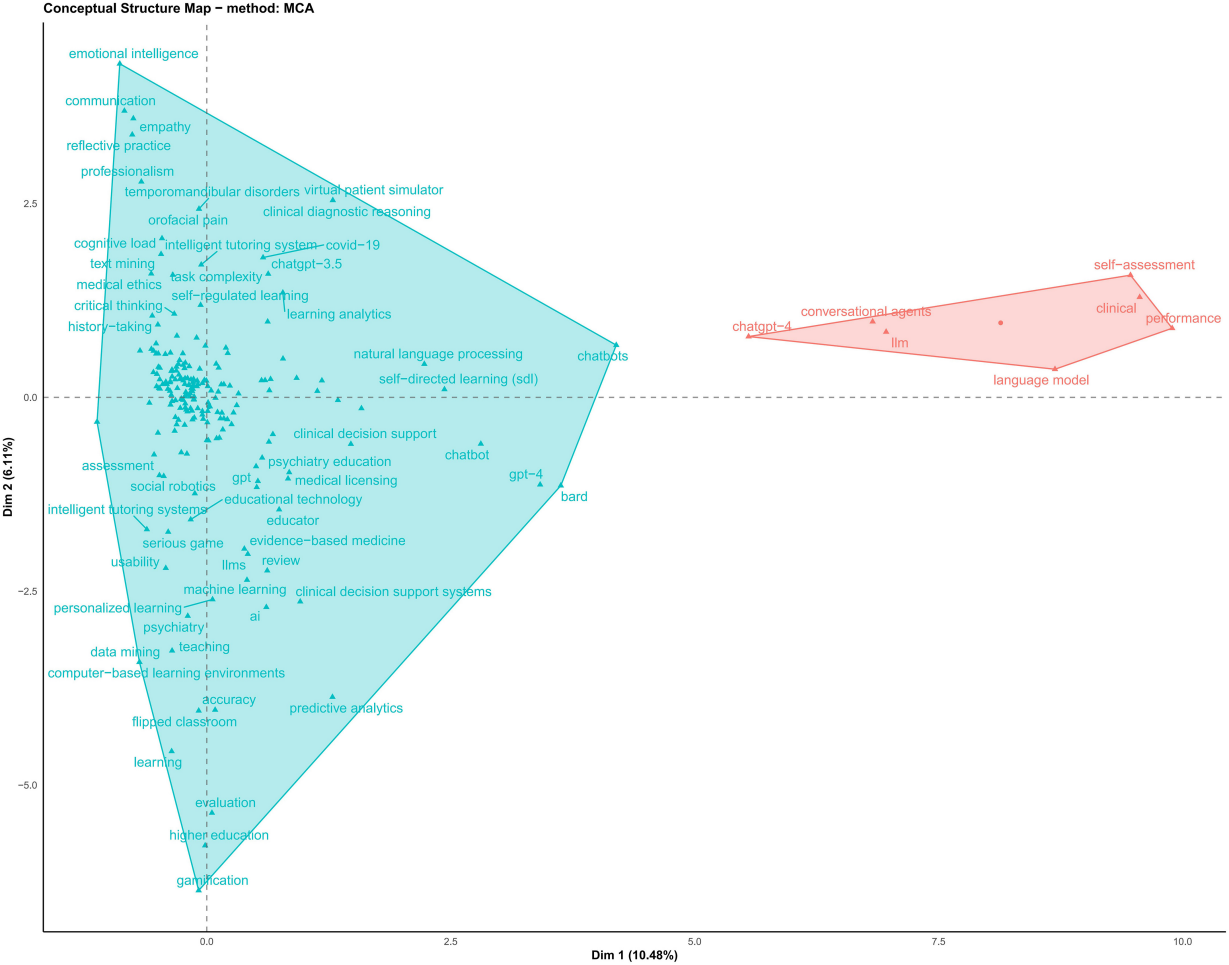
Conceptual structure map (multiple correspondence analysis of author keywords). Dimension 1 (horizontal) distinguishes technology-oriented from pedagogy-oriented research; Dimension 2 (vertical) separates clinical application from educational process themes.

Thematic map analysis (Figure 7) positioned machine learning in the motor themes quadrant (high centrality and density), suggesting a well-developed and central topic. Medical education, clinical reasoning, and self-directed learning were located in the basic themes quadrant (high centrality, lower density), indicating high relevance but relatively lower internal thematic development. Self-regulated learning, natural language processing, and case-based learning appeared as niche themes (low centrality, high density), whereas critical thinking was located near the emerging/declining themes quadrant. Because the thesaurus retained self-directed learning and self-regulated learning as separate constructs, these quadrant positions were interpreted as distinct keyword communities rather than interchangeable labels. The three-field plot (Figure 8) revealed that “artificial intelligence” and “medical education” were the most traversed pathways, with contributions distributed across BMC Medical Education, JMIR Medical Education, and Medical Teacher. Factorial analysis (MCA) extracted two principal dimensions explaining the conceptual structure (Figure 9). Dimension 1 distinguished technology-oriented research (ChatGPT, generative AI, LLMs) from pedagogy-oriented research (education, curriculum, self-directed learning). Dimension 2 separated clinical application themes from educational process themes.

### Co-citation analysis and intellectual foundation

**Figure 10:**
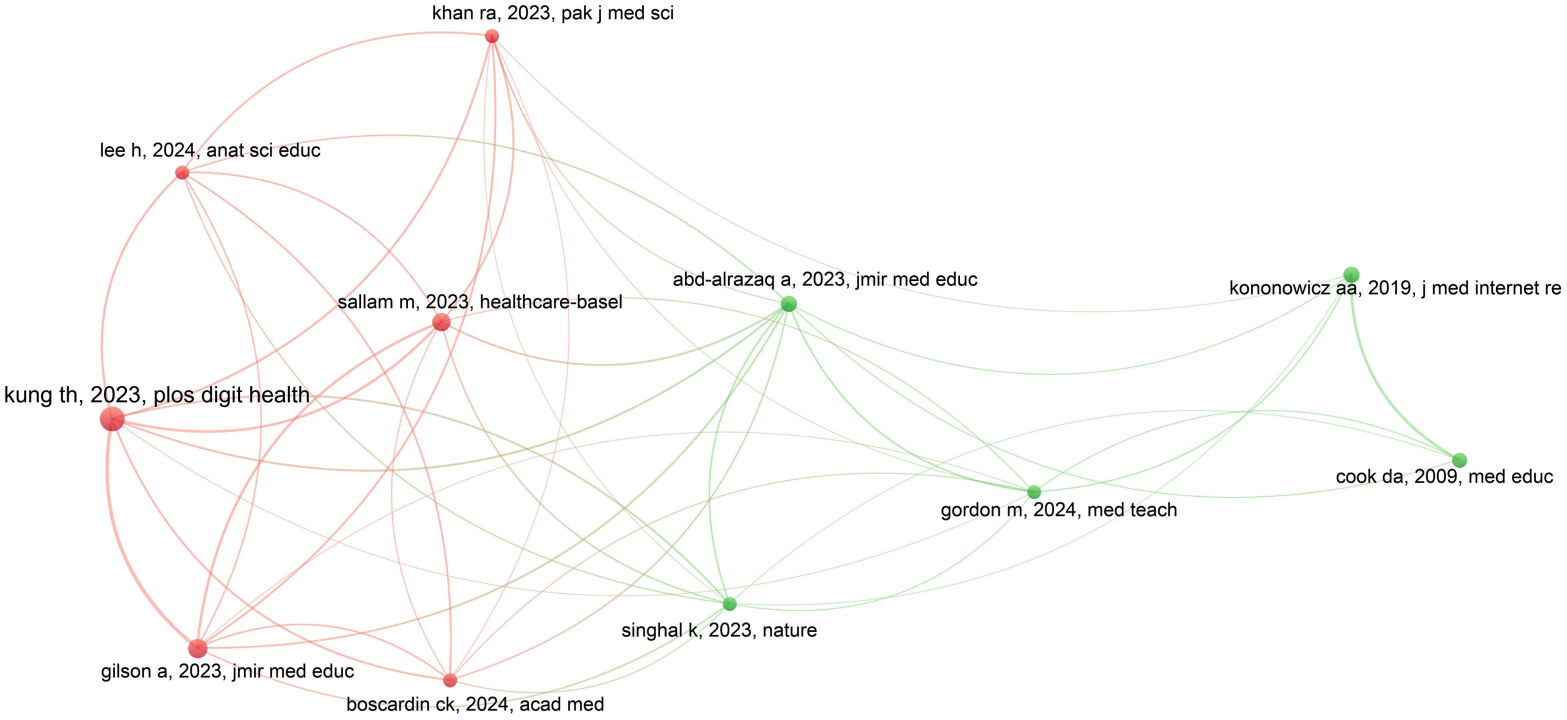
Reference co-citation network (≥20 citations). Colors represent co-citation clusters.

Co-citation analysis of cited references (minimum threshold: 20 citations) identified 11 highly co-cited references forming three major intellectual clusters (Figure 10). The largest cluster (red) comprised core studies on ChatGPT and LLM applications in medical education, anchored by Kung et al.'s 2023 study evaluating ChatGPT's performance on the USMLE (9) as the most frequently co-cited reference (62 co-citations, betweenness centrality = 0.11), alongside Gilson et al. (7), Sallam (12), Lee (5), Khan et al. (20), and Boscardin et al. (6) (Table 3). The second cluster (green) encompassed comprehensive reviews and methodological frameworks for AI in medical education, including Abd-Alrazaq et al. (13), Singhal et al. (8), and Gordon et al.'s BEME Guide No. 84 (11). The third cluster (yellow) contained foundational works on technology-enhanced simulation and virtual patients, including Cook et al.'s landmark systematic review (21) and Kononowicz et al.'s meta-analysis of virtual patient effectiveness (10).

**Table 3:** Top 15 most frequently co-cited references.

Rank	Author(s)	Year	Journal	Co-citations	Centrality
1	Kung TH et al.	2023	PLoS Digit Health	62	0.11
2	Gilson A et al.	2023	JMIR Med Educ	38	0.10
3	Sallam M	2023	Healthcare	32	0.02
4	Abd-Alrazaq A et al.	2023	JMIR Med Educ	25	0.07
5	Khan RA et al.	2023	Pak J Med Sci	21	0.04
6	Gordon M et al.	2024	Med Teach	20	0.03
7	Singhal K et al.	2023	Nature	20	0.01
8	Lee H	2024	Anat Sci Educ	20	0.04
9	Boscardin CK et al.	2024	Acad Med	19	0.04
10	Plackett R et al.	2022	BMC Med Educ	19	0.05
11	Mir Mohammad M et al.	2023	J Adv Med Educ Prof	17	0.01
12	Eysenbach G	2023	JMIR Med Educ	17	0.19
13	Holderried F et al.	2024	JMIR Med Educ	16	0.08
14	Thirunavukarasu AJ et al.	2023	Nat Med	15	0.00
15	Preiksaitis C et al.	2023	JMIR Med Educ	14	0.00

Co-citations, co-citation frequency in the WoS cited-reference dataset used for CiteSpace co-citation analysis; Scopus records were not merged for this specific co-citation network. Centrality, betweenness centrality in the co-citation network. References with ≥20 co-citations are visualized in Figure 10.

### Citation burst analysis

Citation burst detection identified the strongest citation bursts during 2023–2026, confirming generative AI applications as the dominant recent research frontier. References with the strongest burst strength included studies on ChatGPT's performance in medical licensing examinations and empirical evaluations of AI-powered educational interventions.

## Discussion

This bibliometric analysis maps the research landscape of AI integration in PBL- and CBL-based medical education, revealing a field undergoing rapid growth driven by generative AI technologies. The 4.3-fold increase in annual publications from 2022 to 2025, with partial-year 2026 records indicating that this activity was continuing at the time of search, reflects the substantial impact of ChatGPT and other LLMs on this research domain.

A clear inflection point at 2023 marks the year when ChatGPT's public release shifted research emphasis. Pre-2023 research centered on virtual patient simulations and computer-assisted learning systems (10, 21). Post-2023, the keyword landscape expanded to include “ChatGPT,” “large language models,” and “generative AI” as central themes. This trend is consistent with broader patterns in AI-medical education research (15) but is particularly notable in PBL/CBL contexts, where LLMs serve dual functions as both learning tools and potential substitutes for activities such as diagnostic reasoning and literature synthesis (23).

The four thematic clusters identified through keyword co-occurrence analysis highlight distinct research emphases within the field. The nursing and simulation-oriented cluster, centered on nursing education and virtual reality, reflects the substantial attention AI has received in clinical skills training for nursing students, an emphasis that may be driven by the protocol-driven, hands-on nature of nursing curricula where AI-assisted procedural training and feedback systems show immediate applicability (24). The AI and clinical reasoning cluster examines how machine learning and natural language processing can augment aspects of the diagnostic process traditionally facilitated by PBL tutors—arguably the most complex pedagogical function that AI tools are being asked to support. The educational technology cluster, encompassing e-learning, generative AI, and active learning, represents a convergence of AI capabilities with established educational modalities. The LLM-focused cluster, anchored by ChatGPT and large language models, is the most emergent and rapidly expanding: as generative AI tools become more prevalent in clinical practice, PBL curricula are evolving to teach students about AI's capabilities, limitations, biases, and ethical dimensions alongside learning with AI tools (25). The relative separation of these clusters in the network suggests that AI literacy education and AI-assisted pedagogy are developing as parallel rather than integrated streams, a gap that curriculum designers may wish to address.

While the United States and China lead in publication volume, the collaboration network reveals structural differences. The dense European collaborative cluster suggests mature, multidirectional partnerships, whereas the Asia-Pacific cluster anchored by China shows more bilateral connections. The prominence of Turkish researchers (led by Kiyak Y.S. and colleagues) represents an unexpected finding and warrants investigation into the specific regional educational contexts driving this research activity. Morocco's status as the only country in the 33-nation network with zero international co-authorship links (total link strength = 0) may reflect systemic barriers to research collaboration. More broadly, the underrepresentation of low- and middle-income countries (LMICs) mirrors patterns observed in global health education research (22) and is concerning given that AI-driven tools could theoretically help compensate for faculty shortages in resource-limited settings. However, the concentration of research in high-income countries risks developing AI-PBL/CBL solutions optimized for well-resourced environments that may not generalize to contexts with limited digital infrastructure or different cultural approaches to medical education.

Several bibliometric analyses have examined the broader landscape of AI in medical education. For example, recent studies have mapped ChatGPT-related publications across all medical disciplines (15, 16), while other bibliometric reviews have focused on specific AI applications such as virtual reality or machine learning in clinical training. However, these prior analyses did not specifically address the intersection of AI with PBL and CBL methodologies. By narrowing the focus to case-based, small-group learning formats—the predominant pedagogical approach in contemporary medical education—this study provides a more targeted characterization of how AI technologies interact with the specific instructional contexts in which most medical students learn clinical reasoning. The distinct niche-theme position of self-regulated learning, which was not merged with self-directed learning in the thesaurus, highlights a more specific gap in work on learner regulation within AI-supported PBL/CBL contexts.

The co-citation analysis traces an intellectual evolution from virtual patients and technology-enhanced simulation (10, 21) to LLM-driven approaches (9). Thematic map analysis further illuminates this transition: machine learning emerged as a motor theme, whereas clinical reasoning, medical education, and self-directed learning were positioned as basic themes. This suggests that clinical reasoning is highly relevant to the field but remains less internally developed as a distinct thematic cluster than machine learning. The separate positioning of self-regulated learning is notable because it points to learner-regulation processes that are related to, but not identical with, the self-directed learning tradition underpinning PBL (1). The conceptual structure analysis (Figure 9) demonstrates that current literature tends to address AI capabilities or pedagogical implications separately, with relatively few studies integrating both perspectives.

Several limitations should be considered when interpreting these findings. The bibliometric approach captures macroscopic publication trends and network structures but does not evaluate the methodological quality of individual studies, meaning that growth in publication volume does not necessarily equate to growth in high-quality evidence. The analysis was limited to Scopus and WoS, two English-language-dominant databases, which may underrepresent research published in regional journals, particularly from LMICs with relevant educational contexts. The inclusion of CBL alongside PBL, while methodologically justified by their conceptual overlap, may introduce heterogeneity that limits the precision of theme attribution to one pedagogical approach vs. the other. Citation-based metrics are subject to recency bias, which may inflate the apparent impact of more recent publications in a rapidly expanding field. Author and institution disambiguation remains challenging, especially for common Asian surnames; author productivity values for ambiguous names should therefore be interpreted as author-name-string counts rather than verified individual-level counts. Finally, the 2026 data captures only publications indexed through June 2, 2026, and was not extrapolated to estimate full-year output.

Future research should prioritize longitudinal studies that assess whether AI-enhanced PBL translates into measurable improvements in clinical competency and patient outcomes—moving beyond the current reliance on satisfaction surveys and knowledge test scores. Comparative effectiveness research evaluating different AI integration models (supplemental AI tools, AI-as-tutor, and hybrid approaches) would provide evidence to guide resource allocation decisions. Multi-center collaborations that include LMIC institutions should be actively pursued, potentially through targeted funding mechanisms requiring international partnerships. The gap between AI literacy education and AI-assisted pedagogy identified in the thematic clusters suggests that integrated curricular frameworks are needed. As generative AI capabilities continue to advance, the research community should anticipate and study emerging applications such as AI-generated patient cases, real-time tutorial facilitation, and automated competency assessment, ensuring that the evidence base keeps pace with technological development (26).

## Data Availability

The original contributions presented in the study are included in the article/Supplementary material, further inquiries can be directed to the corresponding author.
