# Supplementary material for: Mapping artificial intelligence in problem-based and case-based medical education: a bibliometric analysis (2019–2026)
Source: Front Med (Lausanne). 2026 Jul 30;13:1901004. doi: 10.3389/fmed.2026.1901004 (PMC13470258; doi:10.3389/fmed.2026.1901004)
Supplement: Supplementary file 1 [file Table_1.docx]

# Supplementary Table S1. Complete Literature Search Strategies

**Search date: June 2, 2026**

*Note: The search strings below represent the raw database queries without year or document-type restrictions. Year (2019–2026) and document-type (article, review, conference paper) filters were applied during the screening stage, consistent with the PRISMA 2020 flow diagram (Figure 1).*

## Scopus (Elsevier)

Database URL: https://www.scopus.com

Search interface: Advanced Search

**Raw Search String:**

TITLE-ABS-KEY(("artificial intelligence" OR "machine learning" OR "deep learning" OR "large language model" OR "LLM" OR "ChatGPT" OR "natural language processing" OR "generative AI" OR "GPT" OR "neural network") AND ("problem-based learning" OR "PBL" OR "case-based learning" OR "CBL") AND ("medical education" OR "clinical education" OR "health professions education" OR "nursing education" OR "dental education" OR "medical student" OR "clinical reasoning"))

**Search Fields:**

TITLE-ABS-KEY: Title, Abstract, and Keywords (author keywords plus indexed keywords)

**Filters applied during screening:**

Publication years: 2019–2026 (PUBYEAR > 2018 AND PUBYEAR < 2027)

Document types: Article (ar), Review (re), Conference Paper (cp)

No language restrictions applied at search stage

**Export fields:**

Citation information, Bibliographical information, Abstract & Keywords (33 fields total)

**Records retrieved (raw): 997**

## Web of Science Core Collection (Clarivate)

Database URL: https://www.webofscience.com

Search interface: Advanced Search

**Raw Search String:**

TS=("artificial intelligence" OR "machine learning" OR "deep learning" OR "large language model" OR "LLM" OR "ChatGPT" OR "natural language processing" OR "generative AI" OR "GPT" OR "neural network") AND TS=("problem-based learning" OR "PBL" OR "case-based learning" OR "CBL") AND TS=("medical education" OR "clinical education" OR "health professions education" OR "nursing education" OR "dental education" OR "medical student" OR "clinical reasoning")

**Search Fields:**

TS (Topic): Title, Abstract, Author Keywords, and Keywords Plus

**Filters applied during screening:**

Publication years: 2019–2026

Document types: Article, Review, Proceeding Paper

WoS Indexes: SCI-EXPANDED, SSCI, A&HCI, ESCI

No language restrictions applied

**Export format:**

Plain Text, Full Record and Cited References

**Records retrieved (raw): 619**

## Notes

Total records identified (raw): 1,616

After duplicate removal (DOI-priority, then title-similarity): 1,290

After screening by year (2019–2026) and document type: 735

Final dataset for bibliometric analysis: 735 unique publications

# Supplementary Table S2. VOSviewer Thesaurus File

Purpose: Standardize synonymous and variant terms for keyword co-occurrence analysis in VOSviewer.

Format: Tab-separated values (label → replace by).

| **label** | **replace by** |
| --- | --- |
| chat GPT | ChatGPT |
| chatgpt | ChatGPT |
| ChatGPT | ChatGPT |
| Chat GPT | ChatGPT |
| gpt-4 | ChatGPT |
| GPT-4 | ChatGPT |
| GPT4 | ChatGPT |
| generative artificial intelligence | generative AI |
| generative ai | generative AI |
| AI | artificial intelligence |
| machine learning | machine learning |
| ML | machine learning |
| deep learning | deep learning |
| LLM | large language models |
| large language model | large language models |
| natural language processing | natural language processing |
| NLP | natural language processing |
| problem based learning | problem-based learning |
| PBL | problem-based learning |
| case based learning | case-based learning |
| CBL | case-based learning |
| virtual patients | virtual patient |
| virtual reality | virtual reality |
| VR | virtual reality |
| medical student | medical student |
| medical students | medical student |
| students, medical | medical student |
| nursing student | nursing student |
| nursing students | nursing student |
| students, nursing | nursing student |
| clinical competence | clinical competence |
| clinical reasoning | clinical reasoning |
| self-directed learning | self-directed learning |
| self regulated learning | self-regulated learning |
| self-regulated learning | self-regulated learning |
| controlled study |  |
| questionnaire |  |
| article |  |
| human |  |
| humans |  |
| male |  |
| female |  |
| adult |  |
| young adult |  |
| covid-19 |  |
| coronavirus |  |
| pandemic |  |

*Note: Terms with an empty "replace by" field were excluded from keyword co-occurrence analysis (e.g., generic methodological terms and study design labels that would inflate keyword frequency without contributing to thematic differentiation). Self-directed learning and self-regulated learning were retained as separate constructs; only spelling variants such as "self regulated learning" were standardized to "self-regulated learning."*
